# Prevalence of Computer Vision Syndrome and Associated Factors among Instructors in Ethiopian Universities: A Web-Based Cross-Sectional Study

**DOI:** 10.1155/2021/3384332

**Published:** 2021-10-05

**Authors:** Demisu Zenbaba, Biniyam Sahiledengle, Mitiku Bonsa, Yohannes Tekalegn, Jember Azanaw, Vijay Kumar Chattu

**Affiliations:** ^1^Madda Walabu University Goba Referral Hospital, School of Health Sciences, Department of Public Health, Bale Goba, Ethiopia; ^2^University of Gondar, College of Medicine and Health Science, Department of Environmental Health and Occupational Health and Safety, Gondar, Ethiopia; ^3^Department of Medicine, Faculty of Medicine, University of Toronto, Toronto, ON M5G 2C4, Canada; ^4^Department of Public Health, Saveetha Medical College, Saveetha Institute of Medical and Technical Sciences, Saveetha University, Chennai 600077, India

## Abstract

**Background:**

In this globalized and high-tech era, the computer has become an integral part of daily life. A constant use of computer for 3 hours and more per day can cause computer vision syndrome (CVS), which is one of the leading occupational hazards of the 21^st^ century. The visual difficulties are the most common health problems associated with excessive computer use. Therefore, this study aimed to assess the prevalence and associated factors of CVS among instructors working in Ethiopian universities.

**Methods:**

A web-based cross-sectional study was conducted among 422 university instructors in Ethiopia from February 02 to March 24, 2021. A structured and self-administered questionnaire prepared by Google Forms was shared among instructors through their e-mail addresses, Facebook, and Telegram accounts. Data cleanup and cross-checking were done before analysis using SPSS version 23. A multivariable logistic regression was applied to identify factors associated with CVS using *p* value <0.05 and 95% confidence interval.

**Results:**

Of the total 416 participants, about 293 (70.4%) were reported to have CVS (95% CI: 65.9–74.5%), of which 54.6% were aged 24–33 years. Blurred vision, pain in and around the eye, and eye redness were the main symptoms reported. Working in third-established universities (AOR = 8.44, 95% CI: 5.47–21.45), being female (AOR = 2.69, 95% CI: 1.28–5.64), being 44 years old and above (AOR = 2.73, 95% CI: 1.31–5.70), frequently working on the computer (AOR = 5.51, 95% CI: 2.05–14.81), and sitting in bent back position (AOR = 8.10, 95% CI: 2.42–23.45) were the factors associated with computer vision syndrome.

**Conclusions:**

In this study, nearly seven-tenths of instructors in Ethiopian universities reported having symptoms of computer vision syndrome. Working in third-generation universities, being female, age, frequently working on the computer, and sitting in bent back position were statistically significant predictors in computer vision syndrome. Therefore, optimizing exposure time, addressing ergonomic hazards associated with computer usage through on-the-job and off-the-job training, and making the safety guidelines accessible for all university instructors would be critical to address the problem.

## 1. Background

In this globalized and hi-tech era, the computer has become an integral part of daily life [1]. These devices are deliberated as the necessity of 21^st^ century and being used at workplaces and household level. There has been a rapid increase in computer-related health problems in the current era of prolonged and extensive computer usage [2, 3]. A constant use of computer for 3 hours and more per day can cause computer vision syndrome (CVS), which is defined as a complicated eye and vision difficulty linked to the activities that stress the nearby vision during the use of a computer [4]. This visual difficulty may be caused by a combination of individual visual impediments, poor workstation settings, and insufficient working procedures [5, 6].

Computer vision syndrome is an umbrella term for many eye and environment-related disorders that arise when job-related viewing demands surpass the user's visual capabilities and is characterized by visual symptoms arising from collaboration with a computer monitor and its settings. Ninety percent of the individuals who use the computer for three to four hours daily can develop CVS, and it can be dominant with the symptoms of itching, burning, eye dryness, blurred vision, double vision, and headache that occur during or instantly after the workday [7–10].

Universally, computer vision syndrome is the leading occupational hazard of the 21^st^ century and one of the main public health problems. Global data show that 60 million people are suffering from CVS and one million new cases occur each year. Also, its symptoms affect almost 70% of all computer customers. It is a growing public health concern and contributes significantly to reducing the quality of life and efficiency at the workplace [8, 11, 12]. The economic effect of the visual and musculoskeletal symptoms related to computer usage is great. Underestimating the symptoms that reduce occupational productivity will result in generous financial profit. Visual difficulties are the most frequently occurring health problem related to extreme computer use [13]. Because of barrier inaccessibility and consumption of personal protective equipment, workload, and poor knowledge of ergonomics during computer use, the burden of CVS is high in developing countries such as Ethiopia [14–17].

Earlier studies conducted in different countries showed that the prevalence of CVS ranges from 64% to 90% among computer users [16, 18]. Some of the studies conducted in Ethiopia attempted to figure out the prevalence of CVS and associated factors among computer users in different institutions; however, few individuals are aware of computer vision syndrome, its contributing factors, and simple prevention methods [19, 20]. Factors associated with CVS were commonly categorized as (1) personal factors such as age, poor sitting position, improper viewing distances, medical diseases, taking frequent breaks, and duration of computer usage and (2) the environment which includes improper workstation, poor lighting and computer, imbalance of light between the computer screen and working room surrounding, and poor contrast [21–25].

To the best of our knowledge, the magnitude of CVS and associated factors among instructors in Ethiopia University was not well studied. Therefore, this study was designed to assess the prevalence of CVS and associated factors among instructors working in Ethiopian universities in 2021.

## 2. Materials and Methods

### 2.1. Study Designs, Period, and Settings

A web-based cross-sectional survey was conducted among instructors working in Ethiopian universities from February 02, 2021, to March 24, 2021. Ethiopia is a large, landlocked, and diverse country with more than 90 ethnic and linguistic groups with a population of over 99 million. In Ethiopia, there are ten administrative regions and more than 80 percent of the population lives in rural areas, although there is increased urbanization as workers move from agriculture towards more productive manufacturing and services jobs. A total of 45 universities with 14 first, 23 second, and 8 third established universities are currently giving service in Ethiopia, respectively. The number of academic staff/instructors in Ethiopian universities is estimated to be 32000 [26].

### 2.2. Sampling and Population

The target population was all the instructors working in Ethiopian universities. The study population was all the instructors who use e-mail or social media during the data collection period. The sample size was figured out using a single population proportion formula. Considering 50% proportion, 95% level of confidence, 5% margin of error, and 10% nonresponse rate, we finally obtained 422 sample sizes.

### 2.3. Data Collection Tool

Data were collected through a structured, web-based, and self-administered questionnaire. First, the questionnaires were prepared in English using Google Forms by reviewing earlier studies [23, 25, 27]. The data collection tool includes sociodemographic, ergonomic practice during computer use, and computer vision syndrome items/questions.

### 2.4. Data Collection Methods and Procedures

The prepared Google Form link was shared with the instructors working in Ethiopian universities through their e-mail addresses, Facebook, and Telegram accounts. The Google Form was shared on official social media pages and diverse groups of Ethiopian university instructors' associations/unions to ensure equal representation of participants during the data collection process. The questionnaire became accessible after accepting the terms and conditions of the study. The link to the online Google Form is found at https://docs.google.com/forms/d/e/1FAIpQLSfv5rN6cUJxy6EGp0tW4yUdTuqE8amwi190i7dqCW9htmg5PA/viewform?vc=0&c=0&w=1&flr=0&usp=mail_form_link.

### 2.5. Data Processing and Analysis

The responses of Google Forms were transferred to an Excel sheet and then exported to SPSS 23. Data cleanup and cross-checking were done before analysis using SPSS 23. The frequency, cross-tabulation, charts were used in descriptive analysis. All required assumptions were checked to apply multivariable logistic regression to identify factors associated with computer vision syndrome. In this regard, Hosmer and Lemeshow's model fitness test was used and multicollinearity of independent variables was checked using variance inflation factor (VIF). The variables with a *p* value of <0.20 in the bivariable analysis can be a candidate for the multivariable binary logistic regression. All variables in the multivariable analysis were considered as statistically significant if *p* value is <0.05 with 95% confidence level.

### 2.6. Operational Definitions

#### 2.6.1. Presence of Computer Vision Syndrome (CVS)

In the past one year, if the respondents select at least one of the CVS symptoms such as headache, pain in and around the eye, blurred vision, dry eyes, eye redness, burning sensation, and double vision, the presence of CVS was coded as “yes = 1” if CVS symptoms were reported and “no = 0” if CVS symptoms have not been reported [19, 28].

#### 2.6.2. 20-20-20 Rule for the Eye

After 20 minutes of computer usage, look at something 20 feet away for 20 seconds [29].

#### 2.6.3. First/Second/Third Established University

The 1^st^, 2^nd^, and 3^rd^ oldest universities established in Ethiopia were selected, respectively.

## 3. Results

### 3.1. Sociodemographic Characteristics

A total of 416 respondents have completed the online survey questionnaire with a participation rate of 98.6%. Of these participants, about 144 (34.6%) were from the Oromia region and 219 (52.6%) were from second established universities. The majority (72.4%) of respondents were males, and 227 (54.6%) were within the age group of 24–33 years. Concerning educational status, around 317 (76.2%) of respondents attained up to the second degree or masters and 150 (36.1%) of them were within the 1–5 service years' category (Table 1).

### 3.2. Ergonomic Practices during Computer Utilization

In this study, about 228 (54.8%) respondents were reported to use laptop only. During computer utilization, around 327 (78.6%) respondents adjust the brightness of their computer. About 43% and 83.4% of respondents work on their computer frequently and take regular breaks of 20–60 minutes per day, respectively. Concerning regular sitting position, 159 (38.2%) respondents reported the frequent sitting position with the bent back (Table 2).

### 3.3. Prevalence of Computer Vision Syndrome (CVS)

**Figure 1:**
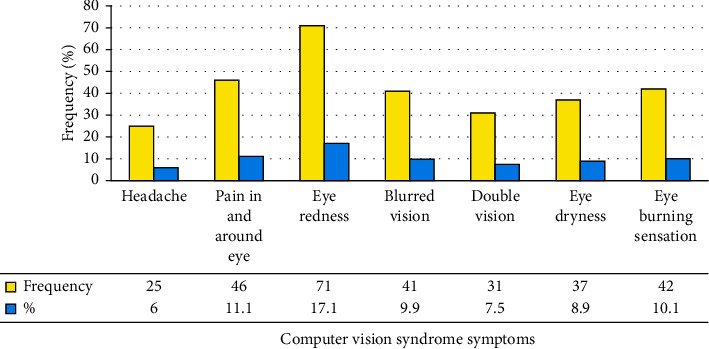
The symptoms of computer vision syndrome mentioned by university instructors in Ethiopia, 2021.

**Figure 2:**
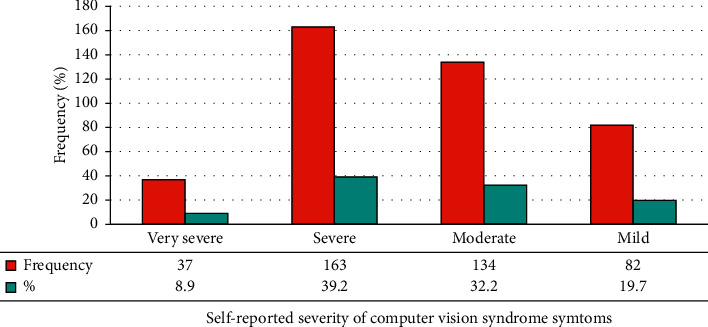
Self-reported severity of computer vision syndrome symptoms among instructors working in Ethiopia universities, 2021.

Among the total participants involved in the study, about 293 (70.4%) were reported to have computer vision syndrome (95% CI: 65.9–74.5%). Commonly reported computer vision syndrome was blurred vision (9.9%), pain in and around the eye (11.1%), and eye redness (8.9%). 134 (32.2%) and 163 (39.2%) respondents reported moderate and severe computer vision syndrome, respectively (Figures 1 and 2).

### 3.4. Factors Associated with Computer Vision Syndrome

The respondents who were working in second and third established Ethiopian universities were nearly seven and eight times more likely to develop computer vision syndrome (CVS) than those working in the first established universities (AOR = 7.34, 95% CI: 5.36–17.54 and AOR = 8.44, 95% CI: 5.47–21.45), respectively. The odds of developing CVS among females were nearly three times higher than males (AOR = 2.69, 95% CI: 1.28–5.64). The instructors within the age category of 44 years old and above were nearly 3 times more likely to develop CVS than their counterparts (AOR = 2.73, 95% CI: 1.31–5.70). The respondents more frequently (always/often) working on their computer were 5.5 times more likely to develop CVS when compared to those working on their computer less frequently (rarely/sometimes) (AOR = 5.51, 95% CI: 2.05–14.81). Regarding ergonomic practices, the instructors who more frequently sit in bent back positions were eight times more likely to report CVS than their counterparts (AOR = 8.10, 95% CI: 2.42–23.45). The odds of having CVS among instructors who did not use eyeglass were 68% less likely than eyeglass users (AOR = 0.32, 95% CI: 0.15–0.67). The instructors who do not know the presence of workplace safety guidelines were nearly six times more likely to develop CVS than their counterparts (AOR = 6.37, 95% CI: 1.68–14.34) (Table 3).

## 4. Discussion

Computer vision syndrome (CVS) is a public health problem associated with computer use. Occupational health and safety regrettably take a backseat most of the time in developing countries such as Ethiopia [29, 30]. This study was designed to assess the prevalence of CVS and associated factors among instructors in Ethiopian universities. In this study, seven out of ten respondents (70.4%) had computer vision syndrome (CVS), with 32.2% moderate and 39.2% severe symptoms, respectively. This finding was similar to that of study conducted in Debre Tabor town, Ethiopia, 69.5% [31], and Malaysia, 68.1% [27]. This consistency might be due to similar characteristics of respondents in computer use. These findings were less than those of a study from Chennai, India, which showed 80.3% [32]. The probable reason for the discrepancy might be either due to sociodemographic variation, study settings, and different duration of exposure to computer electromagnetic radiation. In our study, the most frequent CVS symptoms reported by instructors were redness of the eye and pain in and around the eye, followed by eye burning sensation. These study findings are found to be lower than the findings from Debre Tabor, Ethiopia [31], and India [33]. This discrepancy might be due to sampling size, study participants' age groups, and computer work duration differences.

Computer vision syndrome expressively damages workstation productivity and moderates the quality of life by assigning uncommon strain on the human physical well-being. Regrettably, in this study, some important variables such as duration of university establishment, sex, age, using eyeglass, frequent working, and sitting with bent back position during computer use were factors associated with computer vision syndrome. The instructors working in Ethiopian universities established at the second and third stage were more likely to develop CVS than those working in the first established universities. This observed difference might be due to differences in the implementation of workplace safety guidelines; the most senior universities may have proper infrastructures, services, and supplies that enable instructors to minimize CVS.

The odds of having CVS among females were higher compared to males. This finding was comparable with the study finding in Malaysia, which shows that females have higher odds for CVS when compared to males [27]. This higher rate could be explained by the fact that women more frequently work on the computer and sit in inappropriate positions than males [34]. Our study has revealed that using the eyeglass was significantly associated with CVS; the odds of having CVS among instructors who did not use eyeglass were 68% less likely than eyeglass users. A potential explanation of decreased odds of CVS among those not using eyeglass might be that computer tasks are a type of near work that looks at letters on the screen without shaped tiny dots called pixels.

On the contrary, when the eyeglass is used, it is a little harder to retain the focus images while consistently working on the computer [10]. The respondents within the age category of 44 years and above were nearly 3 times more likely to develop CVS than younger participants. This might be explained by that as age increases, the probability of developing CVS also increases. This finding is inconsistent with the study finding in Malaysia, which shows that the younger age groups were at higher risk of developing CVS [27]. The respondents who were more frequently working on their computers were more likely to develop CVS when compared to those working on their computers less frequently. This might be because a computer emanates electromagnetic radiation or high-energy blue light, which stresses the ciliary muscle in the eye; eventually, a continued exposure to a computer screen causes eye stress. Thus, minimizing the duration of exposure to a computer is important to reduce CVS [10]. This study finding was comparable with that in Debre Tabor, Ethiopia [31], and elsewhere [16, 27].

Regarding ergonomic practices, the respondents who had frequent sitting positions with bending back were more likely to report CVS than their counterparts. This type of practice may reduce the distance between the eye and computer, which exposes them to more electromagnetic radiation emitting from the computer. This study finding was comparable to study findings in Gondar, Ethiopia [25]. The instructors who do not know the presence of workplace safety guidelines were more likely to develop CVS than their counterparts. This might be because they have no probability of using/reading safety guidelines to understand ergonomic hazards and their prevention measures.

### 4.1. Limitation of the Study

This study has a few limitations, such as the ophthalmic checkup was not performed to measure CVS, but was based only on the self-reported symptoms. Since the study used a web-based survey, the respondents were limited to social media and Internet users. The study findings might not reveal the whole country's actual condition due to the underrepresentation of certain universities. The study might not reveal the cause-effect association between dependent and independent variables due to the study's cross-sectional nature.

## 5. Conclusion

Regardless of the above limitation, this study revealed that seven-tenth of instructors in Ethiopian universities reported symptoms of CVS. The most frequent symptoms of CVS reported by instructors were redness of eyes, pain in and around the eye, and burning sensation of the eye. The factors such as university's establishment, sex, age, eyeglass use, types of computer used, workplace safety guidelines, duration of working on the computer, and sitting in bent back position at the computer were identified as associated factors of CVS. Therefore, optimizing the exposure time and minimizing the ergonomic hazards related to computer use through proper job training by developing workplace safety guidelines and making them accessible to all instructors are essential to tackle the problem.

## Figures and Tables

**Table 1:** Sociodemographic characteristics of instructors in Ethiopian universities, 2021 (*n* = 416).

Variables	Frequency	Percent
**Regions**		
Harari	3	.7
Sidama Zone	4	1.0
Gambella	9	2.2
Somali	10	2.4
Dire Dawa	12	2.9
Tigrai	20	4.8
Benishangul-Gumuz	21	5.0
SNNP	56	13.5
Oromia	144	34.6

**Stage of university**		
First generation	133	32.0
Second generation	219	52.6
Third generation	64	15.4

**Sex**		
Male	301	72.4
Female	115	27.6

**Educational status**		
First degree (BSc or BA)	52	12.5
Second degree (Master's)	317	76.2
Third degree (PhD)	47	11.3

**Age**		
24–33 years	227	54.6
34–43 years	162	38.9
44 years and above	27	6.5

**Service years**		
1–5	150	36.1
6–10	129	31.0
11–15	102	24.5
16 and above	35	8.4

**Table 2:** Ergonomic practices and awareness during computer use among instructors in Ethiopian universities, 2021 (*n* = 416).

Items/questions	Frequency	Percent
**Types of computer used**		
Laptop only	258	62.0
Laptop and desktop	153	36.8
Desktop only	5	1.2

**Currently use eyeglass**		
Yes	228	54.8
No	188	45.2

**Do you know the 20-20-20 rule for eyes?**		
Yes	175	42.1
I do not know	241	57.9

**Is your institution having workplace safety guideline?**		
Yes	158	38.0
No	211	50.7
I do not know	47	11.3

**Do you always adjust the brightness of your computer?**		
Yes	327	78.6
No	89	21.4

**How often do you work on your computer?**		
Rarely	22	5.3
Sometimes	57	13.7
Often	179	43.0
Always	158	38.0

**How often is your sitting position upright with bending back?**		
Never	21	5.0
Rarely	61	14.7
Sometimes	137	32.9
Often	159	38.2
Always	38	9.1

**Health break per day**		
20–60 minutes	347	83.4
61–120 minutes	69	16.6

**Table 3:** Bivariable and multivariable logistic regression analysis for computer vision syndrome among instructors in Ethiopian universities, 2021 (*n* = 416).

Variables	Presence of CVS		COR (95% CI)	AOR (95% CI)
	Yes (%)	No (%)		
**Sex**				
Male	213 (70.8)	88 (29.2)	1	1
Female	80 (69.6)	35 (30.4)	1.99 (1.04–3.80)^*∗*^	2.69 (1.28–5.64)^*∗∗*^

**Stage of university**				
First generation	42 (31.6)	91 (68.4)	1	1
Second generation	158 (72.1)	61 (27.9)	7.34 (5.45–20.16)^*∗*^	6.14 (2.45–19.16)^*∗∗*^
Third generation	44 (68.8)	20 (31.3)	6.56 (4.78–18.47)^*∗*^	5.46 (3.78–16.47)^*∗∗*^

**Educational status**				
First degree (BSc or BA)	31 (59.6)	21 (40.4)	1	
Second degree (Master's)	230 (72.6)	87 (27.4)	3.24 (1.36–7.69)^*∗*^	
Third degree (PhD)	32 (68.1)	15 (31.9)	2.71 (0.86–8.58)	

**Age**				
24–33 years	157 (69.2)	70 (30.8)	1	1
34–43 years	121 (74.7)	41 (25.3)	0.36 (0.11–1.16)	0.62 (0.16–2.41)
44 years and above	121 (74.7)	41 (25.3)	2.35 (1.25–4.38)^*∗*^	2.73 (1.31–5.70)^*∗∗*^

**Currently use eyeglass**				
Yes	162 (71.1)	66 (28.9)	1	1
No	131 (69.7)	57 (30.3)	0.41 (0.23–0.73)^*∗*^	0.32 (0.15–0.67)^*∗*^

**Types of computer used**				
Laptop only	183 (70.9)	75 (29.1)	1	1
Laptop and desktop	108 (70.6)	45 (29.4)	0.81 (0.44–1.50)	0.54 (0.27–1.07)
Desktop only	2 (40.0)	3 (60.0)	0.56 (0.36–0.64)^*∗*^	0.42 (0.16–0.81)^*∗∗*^

**Health break per day**				
20–60 minutes	242 (69.7)	105 (30.3)	1	
61–120 minutes	51 (73.9)	18 (26.1)	1.69 (0.80–3.57)	

**Know the 20-20-20 rule**				
Yes	118 (67.4)	57 (32.6)	1	
I do not know	118 (67.4)	57 (32.6)	1.29 (0.84–1.96)	

**Presence of workplace safety guideline**				
Yes	107 (67.7)	51 (32.3)	1	2.58 (1.10–6.06)^*∗∗*^
No	151 (71.6)	60 (28.4)	0.99 (0.54–1.84)	6.37 (1.68–14.37)^*∗∗*^
I do not know	12 (25.5)	35 (74.5)	2.07 (0.71–6.07)	2.58 (1.10–6.06)^*∗∗*^

**Adjust the brightness of the computer**				
Yes	229 (70.0)	98 (30.0)	1	
No	64 (71.9)	25 (28.1)	1.57 (0.79–3.13)	0.199

**Duration of working on the computer**				
Rarely	9 (40.9)	13 (59.1)	1	1
Sometimes	33 (57.9)	24 (42.1)	0.68 (0.12–1.35)	1.20 (0.44–3.31)
Often	132 (73.7)	47 (26.3)	2.06 (1.24–6.54)^*∗*^	3.35 (1.89–8.95)^*∗∗*^
Always	119 (75.3)	39 (24.7)	3.97 (1.65–9.55)^*∗*^	5.51 (2.05–14.81)^*∗∗*^

**Sitting position with bending back**				
Never	13 (61.9)	8 (38.1)	1	1
Rarely	39 (63.9)	22 (36.1)	0.85 (0.30–2.42)	0.76 (0.29–1.99)
Sometimes	90 (65.7)	47 (34.3)	0.25 (0.04–1.44)	0.81 (0.15–4.35)
Often	121 (76.1)	38 (23.9)	2.23 (1.13–4.41)^*∗*^	1.97 (0.90–4.32)
Always	30 (78.9)	8 (21.1)	5.52 (1.83–16.60)^*∗*^	8.11 (2.42–23.45)^*∗∗*^

^*∗*^ *p* < 0.05, crude odds ratio; ^*∗∗*^*p* < 0.05, adjusted odds ratio.

## Data Availability

Data will be made available from the primary author upon reasonable request.

## Abbreviations

CVS: Computer vision syndrome
AOR: Adjusted odds ratio
COR: Crude odds ratio.
